# Structure, Function, and Regulation of the Plasma Membrane Na^+^/H^+^ Antiporter Salt Overly Sensitive 1 in Plants

**DOI:** 10.3389/fpls.2022.866265

**Published:** 2022-03-31

**Authors:** Qing Xie, Yang Zhou, Xingyu Jiang

**Affiliations:** ^1^National Innovation Center for Technology of Saline-Alkaline Tolerant Rice/College of Coastal Agricultural Sciences, Guangdong Ocean University, Zhanjiang, China; ^2^Hainan Key Laboratory for Biotechnology of Salt Tolerant Crops/School of Horticulture, Hainan University, Haikou, China

**Keywords:** Na^+^/H^+^ antiporter, plasma membrane, salt overly sensitive, salt tolerance, Na^+^ extrusion

## Abstract

Physiological studies have confirmed that export of Na^+^ to improve salt tolerance in plants is regulated by the combined activities of a complex transport system. In the Na^+^ transport system, the Na^+^/H^+^ antiporter salt overly sensitive 1 (SOS1) is the main protein that functions to excrete Na^+^ out of plant cells. In this paper, we review the structure and function of the Na^+^/H^+^ antiporter and the physiological process of Na^+^ transport in SOS signaling pathway, and discuss the regulation of SOS1 during phosphorylation activation by protein kinase and the balance mechanism of inhibiting SOS1 antiporter at molecular and protein levels. In addition, we carried out phylogenetic tree analysis of SOS1 proteins reported so far in plants, which implied the specificity of salt tolerance mechanism from model plants to higher crops under salt stress. Finally, the high complexity of the regulatory network of adaptation to salt tolerance, and the feasibility of coping strategies in the process of genetic improvement of salt tolerance quality of higher crops were reviewed.

## Introduction

Soil salinization (mainly NaCl in soil) is one of the main factors restricting global agricultural production, and it seriously threatens the sustainable development of global food production (Dinneny, 2014). If excessive Na^+^ in the soil is absorbed by plant roots, it can seriously damage various physiological metabolic processes, and inhibit plant growth. Compared with halophytes, glycophytes, including most plants and crops, are highly sensitive to salt stress, which further aggravates the harm of salt stress on agricultural production (Ismail and Horie, 2017). Due to the long-term evolutionary processes, higher plants have gradually developed a series of physiological and biochemical response and molecular regulation mechanisms to resist salt stress, in addition to evolving a variety of signal transduction pathways to quickly respond to high salt stress. Some key genes and proteins are involved in a series of regulatory pathways, including signal transduction factors and downstream metabolites (Munns and Tester, 2008; Kurusu et al., 2015; Zhu, 2016). These regulatory factors play key roles in controlling the absorption of salt ions, ion transport, and the growth and development of cells and tissues.

Generally, salt stress in plants is manifested as ionic stress caused by early osmotic stress and Na^+^ accumulation (Munns and Tester, 2008), which further induces secondary stress involving the accumulation of toxic compounds and disruption of nutrient balance. In particular, reactive oxygen species (ROS), such as hydroxyl free radicals, hydrogen peroxide, and superoxide anions will accumulate in plant cells in large quantities (Zhu et al., 2007; Li et al., 2015), seriously damaging the size of plant cell structures and molecular compounds, such as enzymes, DNA, and lipids (Golldack et al., 2014; Singhal et al., 2021). Since plants need to maintain water potential lower than that of the soil to ensure that the cells can absorb enough water to maintain growth, plants under salt stress usually reduce water loss or increase water absorption to alleviate damage caused by osmotic stress. At the same time, plants have evolved mechanisms to avoid high salt stress and maintain the concentration of Na^+^ in cells below the toxic level (Zhu, 2002; Munns and Tester, 2008). To respond to the harm caused by salt stress, plants can transport Na^+^ out of the cytosol through Na^+^ efflux by the plasma membrane Na^+^/H^+^ antiporter salt overly sensitive 1 (SOS1; Parida and Das, 2005; Roy et al., 2014) and Na^+^ compartmentalization by the tonoplast Na^+^/H^+^ antiporter Na^+^/H^+^ exchanger (NHX; Apse, 1999; Yokoi et al., 2002; Apse et al., 2003), increase intracellular osmotic adjustment substances (Munns and Tester, 2008; Munns and Gilliham, 2015) and restrict long-distance transportation of Na^+^ (Shi et al., 2002). Recently, the tonoplast NHX1 and NHX2 proteins were proved to function in K^+^ and pH homeostasis, other than in salt tolerance (Bassil et al., 2011; Barragan et al., 2012; van Zelm et al., 2020). So SOS1 protein excreting Na^+^ may be the most important determinant in improving plant salt tolerance.

Salt overly sensitive 1 is the only known plasma membrane Na^+^ efflux protein so far, and its role in controlling ion homeostasis has been identified through genetic, biochemical, and physiological analyses (Qiu et al., 2002; Shi et al., 2002; Quintero et al., 2011) in both monocots and dicots (Martínez-Atienza et al., 2007; Xu et al., 2008; Quintero et al., 2011). The SOS signaling transduction pathway, consisting of SOS1, Salt Overly Sensitive 2 (SOS2)/CIPK24 or CIPK8 and Salt Overly Sensitive 3 (SOS3) or calcineurin B-like protein 10 (CBL10)/SOS3-like calcium binding protein 8 (SCABP8), has been characterized in glycophytes, such as *Arabidopsis* (Quintero et al., 2011; Yin et al., 2020), poplar (Tang et al., 2010), rice (Martínez-Atienza et al., 2007), and halophytes such as *Thellungiella salsuginea* (Oh et al., 2009). Besides, SOS1 was regulated by radical-induced cell death regulator (RCD1; Katiyar-Agarwal et al., 2006) and phospholipase D (PLD) signaling pathway under salt stress (Yu et al., 2010). Therefore, the regulatory mechanism of Na^+^ efflux by SOS1 protein is more sophisticated than our understanding.

The function and application of single SOS1 protein or other SOS pathway components in response to salt tolerance have been investigated in many plant species. However, it is becoming increasingly difficult to significantly improve plant salt tolerance only relying on single gene. In this study, we reviewed the structure, function of SOS1 protein, especially its regulatory mechanism, and gave some suggestions for breeding salt tolerant crops in the future.

## Kinetics and Energetics of Na^+^ Absorption and Discharge

When plants are exposed to an environment with high concentrations of NaCl, root tissues accumulate a higher concentration of Na^+^ within first 2 min, and 10 min later, Na^+^ is detected to flow out of the root tissues (Bose et al., 2015). This indicates that plants are rapidly able to sense high external concentrations of Na^+^ and trigger the downstream salt stress responses. Under conditions of high soil salinity, Na^+^ enters plant cells primarily through passive transportation due to the high concentration of external Na^+^. The electrophysiological characteristics of Na^+^ flowing into root epidermis and cortex cells and the characteristics of Na^+^ flowing into intact root cells indicate that Na^+^ inflow in plants is likely to be passively mediated through non-selective cation channels (NSCC; Tester and Davenport, 2003; Demidchik and Maathuis, 2007); however, this non-selective transport (including monovalent cations and divalent cations, such as Ca^2+^ and Mg^2+^) is actually beneficial to nutrient absorption by plants. Na^+^ enters the plant cell and crosses the endothelial layer, enters the central vascular cylinder (stele), loads from the pillar cells to the ducts and tracheids responsible for long-distance transport in the xylem, and is then transported to above-ground tissues *via* transpiration flow (Munns and Tester, 2008). The accumulation of Na^+^ in above-ground tissues is due to the Na^+^ transport process in different organs and cell types (Dinneny, 2014). In some plants, ion channels in the high-affinity K^+^ channel (HKT) family may be involved in the Na^+^ influx process, but in *Arabidopsis*, HKT is not the main contributor to Na^+^ entering the cell (Demidchik and Tester, 2002). In addition, many NSCCs are permeable to Ca^2+^. Therefore, during periods of salt stress, NSCC may be related to Ca^2+^ nutrition and signal transduction, in addition to the absorption of Na^+^ by plants. At the same time, Ca^2+^ shows a strong blocking effect on Na^+^ currents mediated by NSCC (Demidchik and Maathuis, 2007). This potentially explains, in part, why the salt tolerance of plants can be improved by external application of Ca^2+^.

Based on existing research, the specific transporter responsible for Na^+^ absorption has not been clearly identified, and the entry of Na^+^ into the cell through the cell membrane involves not only weak voltage-dependent NSCCs, but also HKTs, low-affinity K^+^ channels (AKT1), non-selective outward ion channels, and non-voltage-gated ion channels (VICs), among others (Blumwald et al., 2000; Tuteja, 2007). All of these ion channels and transporters can regulate the influx of Na^+^ and K^+^ from outside the cell into the cytoplasm, and their activity can affect cytoplasmic levels of Na^+^ and K^+^, ultimately affecting the degree of cell damage and the absorption of nutrients. Through evolution, higher plants have established a series of regulatory mechanisms to maintain a suitable dynamic ion balance under stress and regulate normal growth.

Exhausting excess cytoplasmic Na^+^ through the cytoplasmic membrane is one way plant cells can relieve Na^+^, representing an important method of toxicity excretion (Deinlein et al., 2014). Through genetic screening of mutants sensitive to salt stress, a Ca^2+^-dependent Na^+^ efflux mechanism has been discovered and characterized. This regulatory mechanism was named the Salt Overly Sensitive (SOS) pathway. SOS1 is the main transporter involved in this process (Zhu et al., 1998; Hasegawa et al., 2000; Jiang et al., 2013). From a thermodynamic point of view, SOS1-mediated Na^+^ excretion from the cytoplasm under salt stress is an active transport process. In plants, this energy-consuming Na^+^ transport process is accompanied by the activity of H^+^-ATPase, which forms a H^+^ electrochemical potential gradient across the plasma membrane to drive for Na^+^ excretion (Blumwald et al., 2000; Zhou et al., 2015; Fan et al., 2019). Under normal conditions, the plasma membrane H^+^-ATPase is in an inhibited state and requires minimal activity to maintain cellular pH at steady levels (Yang et al., 2019). At the same time, the proton pump can cause plasma membrane hyperpolarization, reduce the polarity of the plasma membrane under salt stress, inhibit Na^+^/K^+^ channel protein activity, and reduce K^+^ efflux. Studies have shown that the free unsaturated fatty acids in *Arabidopsis* root cells bind directly to the C-terminus of the plasma membrane H^+^-ATPase AHA2 under salt stress to activate plasma membrane H^+^-ATPase activity (Han et al., 2017). The fatty acids accumulated in plants are also necessary for the salt resistance process (Zhang et al., 2012).

## Plasma Membrane Na^+^/H^+^ Antiporter

### Structure and Phylogeny

**Figure 1:**
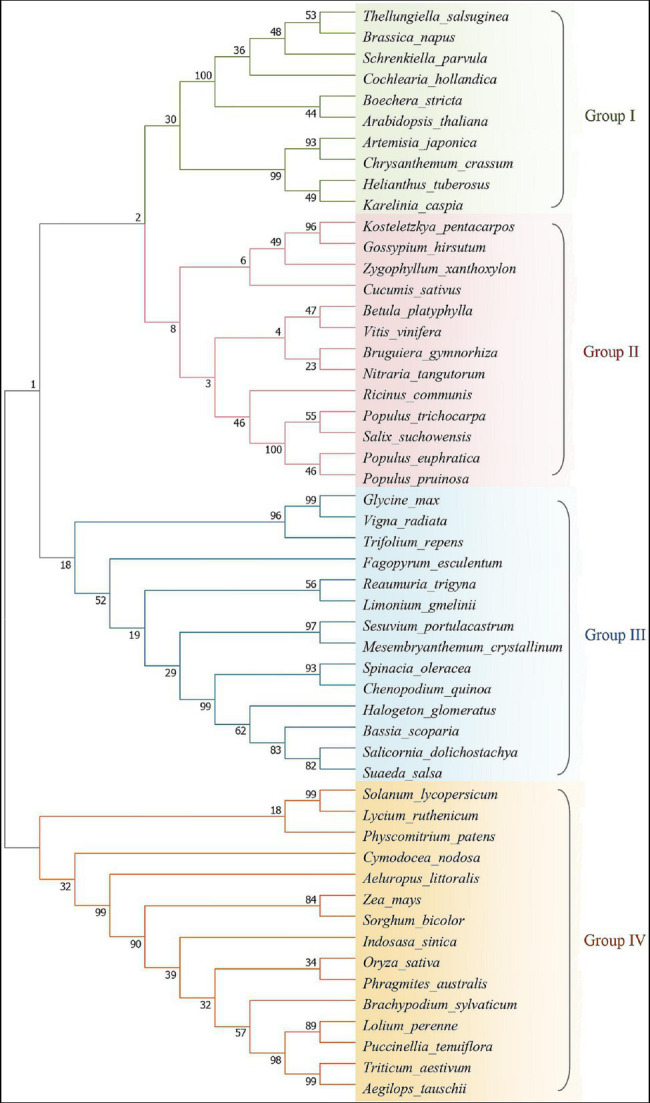
Phylogenetic relationship analysis of salt overly sensitive 1 (SOS1) reported in plants. The amino acid sequences were aligned and the tree of sequence relationships generated using MEGA7. Classification was based on protein homology analysis, and groups of classifications were partitioned by different colors, each color representing the affinity of the plasma membrane Na^+^/H^+^ antiporter SOS1 between different species. The plant sequences are known by their GenBank accession numbers and the accession numbers of these plant sequences as follow: *Glycine max* (AFD64746), *Karelinia caspia* (QOS02253), *Fagopyrum esculentum* (AVZ44366), *Lolium perenne* (AAY42598), *Arabidopsis thaliana* (OAP11543), *Nitraria tangutorum* (AGW30210), *Reaumuria trigyna* (AGW30208), *Bruguiera gymnorhiza* (ADK91080), *Physcomitrium patens* (CAM96566), *Solanum lycopersicum* (NP_001234698), *Trifolium repens* (AWS33581), *Bassia scoparia* (BAX01505), *Halogeton glomeratus* (AMK51995), *Chrysanthemum crassum* (BAR88076), *Artemisia japonica* (ALH21662), *Spinacia oleracea* (CDL70805), *Salicornia dolichostachya* (CDL70804), *Triticum aestivum* (AIA08676), *Aeluropus littoralis* (AEV89922), *Limonium gmelinii* (ACF05808), *Chenopodium quinoa* (ABS72166), *Gossypium hirsutum* (AKN19929), *Vitis vinifera* (NP_001268140), *Oryza sativa* (ATU90113), *Cucumis sativus* (NP_001292661), *Lycium ruthenicum* (QBF58650), *Brassica napus* (ACA50526), *Populus pruinosa* (AQN76710), *Betula platyphylla* (ALV66191), *Populus euphratica* (AQN76692), *Ricinus communis* (APR62626), *Helianthus tuberosus* (AGI04331), *Suaeda salsa* (AHJ14584), *Boechera stricta* (AHB86984), *Vigna radiata* (AGR34307), *Indosasa sinica* (AGB06353), *Sesuvium portulacastrum* (AFX68848), *Cochlearia hollandica* (AFF57539), *Aegilops tauschii* (CAX83737), *Mesembryanthemum crystallinum* (ABN04858), *Cymodocea nodosa* (CAD20320), *Salix suchowensis* (KAG5240570), *Kosteletzkya pentacarpos* (AIS92905), *Zea mays* (QOI16623), *Schrenkiella parvula* (ADQ43186), *Zygophyllum xanthoxylon* (ACZ57357), *Brachypodium sylvaticum* (ACO87666), *Phragmites australis* (BAF41923), *Populus trichocarpa* (AVA17745), *Puccinellia tenuiflora* (ABO32636), and *Sorghum bicolor* (XP_002443674).

Na^+^/H^+^ antiporter is an inner membrane protein that can exchange Na^+^ and H^+^ through the cell membrane or cytoplasmic inner membrane. They are essential for transporting Na^+^, adjusting pH, and maintaining cytoplasmic homeostasis. They are targeted by human drugs and also represent the basis of salt tolerance in plants (Hunte et al., 2005). The plant Na^+^/H^+^ antiporter SOS1 and other NHX proteins belong to the Cation Proton Antiporter (CPA) protein family (Shi et al., 2000; Brett et al., 2005). They exhibit high homology to the Na^+^/H^+^ antiporters of bacteria and some eukaryotes. According to the SOS1 sequences of reported plant species, homology comparison and phylogenetic tree analysis were conducted (Figure 1). More distant homology with *Arabidopsis*, *Oryza sativa* is closely related to *Triticum aestivum* and belongs to the cluster 4, which also includes *Solanum lycopersicum* and *Lycium ruthenicum*, *Zea mays*, and *Sorghum bicolor*. The *Glycine max*, *Fagopyrum esculenturn*, and *Chenopodium quinoa* belong to the third cluster, but the homology among the three is not very closely. The *Sesuvium portulacastrum* and *Mesembryanthemum crystallinum* have 99% homology, and the *G. max* has high homology with *Vigna radiate* as expected. In group number 2, there are *Populus euphratica*, *Gossypium hirsutum*, *Bruguiera gymnorhiza*, *Chrysanthemum crassum*, etc., and the halophytes are distributed in each cluster. In short, the homology between each cluster indicates that the differentiation may occurred early during evolutionary, therefore the evolution of salt tolerance mechanism from model plants to higher plants is specific to some extent.

**Figure 2:**
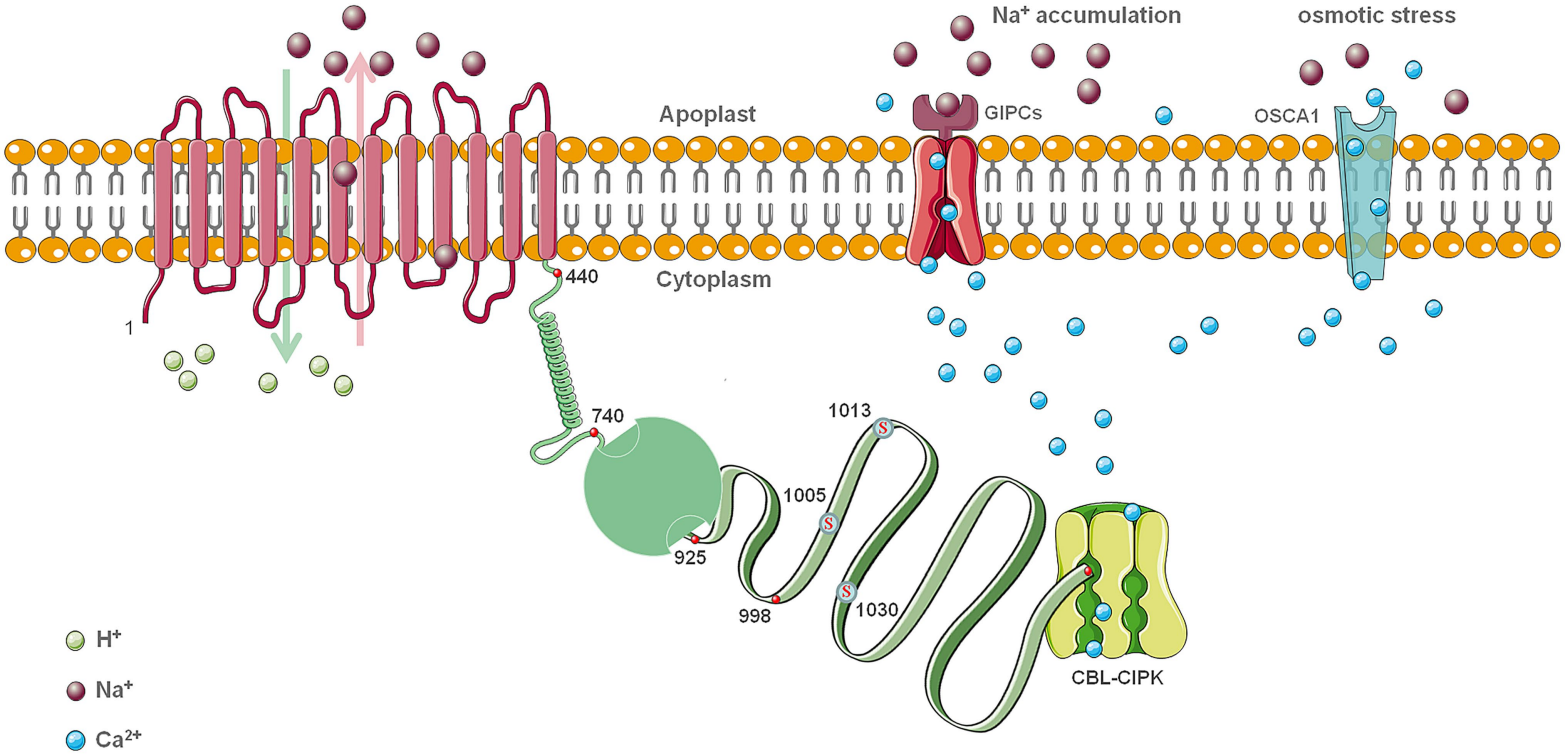
The model structure of SOS1 in salt stress. Based on an analysis of *Arabidopsis*, the assignment of the 12 transmembrane domains (TMDs; 1–440) was assumed evenly distributed. The region 742–925 is functional activation area and enclosed by the C-terminus (998 –1,146) in the absence of salt stress, and the folded region is the disordered non-conservative region from 925 to 997. The boundaries of these regions should not be considered as consistent for all species. The red mark at the end of C-terminus represents DSPS, a conservative phosphorylation site recognized by the calcineurin B-like protein (CBL)-CIPK protein kinase complexes. Other three serine conservative phosphorylation sites at the C-terminus are also highlighted in red color. Glycosyl inositol phosphorylceramide sphingolipids (GIPCs) function as a Na^+^ receptor and Ca^2+^ channel under salt stress. OSCA1 functions as a Ca^2+^ channel under osmotic stress. This is still a reference model that needs experimental verification.

Structural results show that SOS1 is similar in structure to the Na^+^/H^+^ antiporter NhaA of *Escherichia coli* and NhaP1 from Methanogens. Both exist as dimers on the plasma membrane and consist of 12–13 transmembrane helices (Figure 2; Baumeister and Steven, 2000). The structure of the crystalline NhaA monomer is natural. Many helices do not change their conformation as pH changes. The monomer is the functional unit of NhaA, and the dimer plays a vital role in antiporter stability under extreme stress conditions. Na^+^/H^+^ antiporters are usually strictly regulated by pH, and their activation is caused by changes in pH. Studies have shown that subtle rearrangement of the two transmembrane helical structures in response to PH may be related to Na^+^ transport (Appel et al., 2009). In contrast to the structures of other ion transporters, NhaA exhibits a folding structure in the middle of the membrane that creates a delicately balanced electrostatic environment. This structure is likely to be a necessary condition for the binding and transport of Na^+^ and H^+^ ions (Hunte et al., 2005). Bioinformatics analysis based on secondary structure, globular prediction, and sequence conservation indicates that the carboxy-terminal part of the cytoplasmic portion of SOS1 is composed of three domains (Cole et al., 2008). The first one spans residues 526–740 and is almost identical to the cytoplasmic domain of AtNHX8, which is a homolog of the *Arabidopsis* Na^+^/H^+^ antiporter involved in lithium transport (An et al., 2007). The second domain is shown as a cyclic nucleotide binding domain (CNBD), and the third domain, which is connected to the second domain by a disordered region, is expected to include residue 998 through the C-terminus (Figure 2; Núñez-Ramírez et al., 2012). The carboxy-terminal cytoplasmic region of SOS1 forms two different regions with higher density thresholds. These domains will be rearranged and activated. Therefore, the regulatory region should be flexible and may be partially disordered. The difference between these transporters is mainly reflected in the modes of interaction at the dimerization interface (Goswami et al., 2011). The above studies provide a structural framework for the SOS1 protein, and molecular genetic studies have demonstrated that the cytoplasmic portion of SOS1 can be functionally divided into an activation region and an inhibitory region. With additional research, a model of SOS1 regulation has been proposed. The interaction between these two parts can allosterically regulate the activity of the Na^+^/H^+^ antiporter SOS1 (Quintero et al., 2011). In fact, because SOS1 connects spatially remote regulatory and functional domains, it serves the biological function of the neutral Na^+^/H^+^ antiporter (Shi et al., 2002). Transmission of information from the plasmin domain to the transmembrane domain (TMD) through the dimer structure of the antiporter provides a stable, large spherical docking platform that integrates signals from different pathways and regulates Na^+^ transport. The detailed mechanisms of such processes remain to be further studied or corroborated by higher resolution structural biology techniques.

### Function

Although the molecular mechanism by which plants limit the rate of Na^+^ absorption under salt stress is still unclear, researchers have explored the mechanism by which plant cells rapidly expel Na^+^ in response to salt stress. In recent years, the SOS1 gene has been discovered in many plants, such as halophytes (Oh et al., 2009), *O. sativa* (Martínez-Atienza et al., 2007), *T. aestivum* (Xu et al., 2008), *S. lycopersicum* (Olias et al., 2009), *Physcomitrium patens* (Fraile-Escanciano et al., 2010), *Chrysanthemum crassum* (Song et al., 2012), and the woody plant *Populus tomentosa* (Tang et al., 2010), *Spinacia oleracea* (Zhao et al., 2021a), among others (Brindha et al., 2021; Figure 1). Among known SOS1 proteins in plants, the *Arabidopsis thaliana* SOS1 protein is the first plasma membrane protein to have been comprehensively characterized as a Na^+^/H^+^ antiporter from physiological, biochemical, and molecular perspectives (Wu et al., 1996; Quintero et al., 2011). Based on DNA sequence analysis of the *Arabidopsis SOS1* gene, *AtSOS1* encodes 1,146 amino acids, containing 23 exons and 22 introns. It is currently the longest known Na^+^/H^+^ antiporter. SOS1 is located on the plasma membrane and is a highly conserved part of the plant salt tolerance pathway. Under salt stress, expression of SOS1 is increased in rice and *A. thaliana*; transcript levels are increased in wheat plants exposed to high-salt environments (Shi et al., 2000; Martínez-Atienza et al., 2007; Xu et al., 2008); *Thellungiella salsuginea*, a halo-relative of *A. thaliana*, exhibits higher SOS1 mRNA levels than *A. thaliana* under salt stress (Oh et al., 2009), and *Arabidopsis* SOS1 knockout plants are highly sensitive to salt stress (Wu et al., 1996; Zhu et al., 1998). The above results indicate that SOS1 is a crucial protein involved in plant response to salt stress. Furthermore, expression of endogenous SOS1 or homologous SOS1 from other plants can rescue the salt-sensitive phenotype of *sos1 Arabidopsis* mutants (Shi et al., 2000; Martínez-Atienza et al., 2007). Under NaCl treatment, *A. thaliana* with SOS1 overexpression exhibited better growth ability than wild-type plants (Shi et al., 2003). The expression of wheat SOS1 promoted growth of transgenic tobacco (*Nicotiana tabacum*) under NaCl treatment (Zhou et al., 2016). These findings suggest that SOS1 plays an important role in maintaining cytoplasmic Na^+^ levels, enabling plants to become salt tolerant.

In addition, SOS1 is also involved in the long-distance transport of Na^+^ from roots to shoots, oxidative stress, and intracellular pH balance (Qiu et al., 2002; Shi et al., 2002; Katiyar-Agarwal et al., 2006; Oh et al., 2010; Quintero et al., 2011; Feki et al., 2014). The salt phenotype of the *sos1* mutant is the most sensitive, indicating that SOS1 is located in the most downstream portion of the SOS pathway and is involved in regulating plant salt stress response (Halfter et al., 2000; Shi et al., 2000; Qiu et al., 2002; Lin et al., 2009; Yin et al., 2020). Biochemical analysis showed that SOS1 was mainly expressed in the epidermal cells of the root tip and the parenchyma cells at the xylem/symbiont boundary of roots, stems, and leaves. Under mild stress conditions (25 mM NaCl), the aerial portion of the *sos1* mutant accumulated fewer sodium ions than the wild type, while under severe stress conditions (100 mM NaCl), the aerial portion of the *sos1* mutant accumulated significantly more sodium ions than the wild type (Shi et al., 2002). These results indicate that SOS1 plays an important role in the long-distance transport of sodium ions.

### Regulation

#### Na^+^ Induction and Calcium Transients

When faced with salt stress, plants first need to sense a salt stress signal, and then activate a signal transduction pathway to respond to the stress environment. In order to effectively cope with salt stress, plants have evolved components that sense osmotic stress and Na^+^ stress. Although both of these stresses can induce an increase in the concentration of free cytoplasmic calcium ions, plants respond to inchoate osmotic stress (rapid) and later poisonousness of Na^+^ (slow) in significantly different ways (Munns and Tester, 2008). Through analysis of the *Arabidopsis* transcriptome, it has been shown that both salt stress and osmotic stress can regulate expression of many stress response genes in plants. Among 932 upregulated genes and 367 downregulated genes induced by salt stress, 435 and 154 genes, respectively, overlapped with 1,118 upregulated and 364 downregulated by osmotic stress (Sewelam et al., 2014). Transcriptome analysis of bread wheat leaves also showed that salt stress activated the expression of other stress response genes in plants (Amirbakhtiar et al., 2021). These indicate that salt stress may be sensed by osmotic stress and Na^+^ receptors, triggering permeation and ion signal transduction. It has been reported that OSCA1 is a newly discovered osmotic receptor in plants. The *osca1* mutant exhibits lower cytosolic Ca^2+^ as well as weak transpiration and root development in response to external osmotic stress than the wild type (Yuan et al., 2014). Therefore, OSCA1 was identified as a membrane protein that can form a highly osmotically regulated Ca^2+^ channel. Glucuronosyltransferase for glycosyl inositol phosphorylceramide sphingolipids (GIPCs) in plasma membrane can sense ionic stress signaling *via* binding with Na^+^ and gate Ca^2+^ influx channels under salt stress, such that Ca^2+^ concentration increases in cytosol, so DIPCs may act as a receptor of Na^+^ stress (Jiang et al., 2019). Another study found that AtANN4 can act as a new type of Ca^2+^ sensor that participates in the osmotic transporter (Ma et al., 2019). Heterologous expression of AtANN4 can promote Ca^2+^ influx and is regulated by SCABP8 and CIPK24. AtANN4 mediates the increase of cytosolic Ca^2+^ level under multiple pressures, indicating that AtANN4 may act as a Ca^2+^ permeable transporter or a cofactor for Ca^2+^ channels, but does not produce specific calcium signals (Liao et al., 2017). It can be inferred that AtANN4 may play a role in the early stages of plant salt stress by generating a calcium signal response mechanism.

Salt stress leads to an increase in the concentration of Ca^2+^ in plant cells. The calcium binding proteins SOS3 (Quan et al., 2007; Zhu, 2016) and SCaBP8/CBL10 can recognize and decode elevated Ca^2+^ signals (Liu and Zhu, 1998; Ishitani et al., 2000; Quan et al., 2007; Zhu, 2016), Ca^2+^-bound SOS3 and SCABP8 bind and activate the serine/threonine protein kinase SOS2 and recruit it to the plasma membrane (Halfter et al., 2000; Quintero et al., 2002; Quan et al., 2007; Lin et al., 2009). SOS2 activates activity of the plasma membrane Na^+^/H^+^ antiporter SOS1 through phosphorylation (Qiu et al., 2002; Lin et al., 2009). Every member of the SOS signaling pathway plays an important role in resisting salt stress. Studies performed in a heterologous yeast expression system demonstrate that expression of SOS1 can endow salt-sensitive yeast with a certain salt tolerance. When SOS signal pathway members SOS2 and SOS3/SCaBP8 are expressed simultaneously, the tolerance of yeast to salt stress is significantly improved (Quintero et al., 2002; Quan et al., 2007), indicating that members of the SOS signaling pathway are necessary for the process of salt stress response. As calcium signal sensing and decoding proteins, SOS3 and SCaBP8 are essential for activation of the SOS signal pathway. The SOS3 gene deletion mutant has an obvious salt-sensitive phenotype, and the addition of external Ca^2+^ can restore the salt-sensitive phenotype to a certain extent (Liu and Zhu, 1998). The amino-terminus of SOS3 protein contains a sequence characteristic of myristoylation. Genetic phenotype analysis revealed that SOS3 with the myristoylation modification can restore the salt-sensitive phenotype of sos3, while non-myristoylated SOS3 cannot restore this salt-sensitive phenotype. Subsequent research found that myristoylation of the amino terminus of SOS3 can help SOS3 efficiently localize to the plasma membrane (Ishitani et al., 2000). SCaBP8 and SOS3 have similar functions, and both can interact with SOS2 and recruit it to the plasma membrane to perform functions. Both have a certain degree of functional redundancy in addition to their own independent functions. Activity of SCaBP8 is essential for vegetative growth under salt stress; it can act independently of the SOS pathway and can regulate the reproductive growth of plants under salt stress (Monihan et al., 2016). Salt stress can induce increased expression of *SCaBP8*, but the expression level of *SOS3* is not obviously changed, potentially due to differences in expression position between the two genes. Genetic phenotype analysis shows that the salt-sensitive phenotype of *sos3* is mainly apparent in the roots, while the salt-sensitive phenotype of *scabp8* manifests as an obvious inhibition of shoot growth (Quan et al., 2007; Zhu, 2016).

#### Auto-Inhibition and Phosphorylation Regulation

In response to adversity, plants typically convert stress signals into specific protein interactions that can activate the biological functions of a series of effecter proteins. The regulation process is complex and precise, and regulated proteins or kinases exhibit a corresponding inhibitory state to maintain cell homeostasis. Protein phosphorylation is the main way by which activity of the plasma membrane Na^+^/H^+^ antiporter SOS1 is regulated. Molecular structure analysis determined that the downstream region of the amino-terminal TMD of SOS1 contains a functional domain and the carboxyl end of the self-inhibitory domain, and the portion is connected by a connecting region. The self-inhibitory domain and the connecting domain are essential for the regulation of SOS1 activity (Quintero et al., 2002). Under normal conditions in *Arabidopsis*, both SOS1 and SOS2 are in a state of self-inhibition. The SOS1 self-inhibitory region inhibits its own activity by interacting with the functional domains, maintaining a low level of SOS1 activity (Schachtman and Liu, 1999; Shi et al., 2000; Quintero et al., 2002; Núñez-Ramírez et al., 2012). When a salt stress signal is sensed, the SOS2 protein kinase and its complex proteins bind to conserved serine phosphorylation sites at positions 1,136 and 1,138 of the SOS1 self-inhibitory domain, releasing the inhibition of the SOS1 self-inhibitory domain on functional domain activity. This process releases self-inhibitory effects and activates SOS1 (Quintero et al., 2011; Jarvis et al., 2014). Mutation of the serine site phosphorylated by SOS2 in the SOS1 sequence to an alanine residue, which mimics the non-phosphorylation state of SOS1, demonstrated that mutations in the conservative phosphorylation site Ser1136/Ser1138 also reduce tolerance to salt stress (Quintero et al., 2011; Yin et al., 2020). The mutation of serine residues at positions 1,136 and 1,138 into aspartic acid residues, mimicking phosphorylation, can enhance binding to SOS2 (Quintero et al., 2002). Upon deletion of the carboxyl terminus downstream of the functional domain of SOS1, the Na^+^/H^+^ antiporter activity of SOS1 is constitutively activated (Guo et al., 2001; Gong et al., 2002; Zhou et al., 2016). In addition, there may also be an important serine residue in the SOS1 linking domain that plays a key role in activity modulation (Figure 2). The absence of the SOS1 linking domain will affect regulation by protein kinases, leading to the partial loss of SOS1 activity (Quintero et al., 2002; Zhou et al., 2016). SOS2 also exists in a self-inhibitory form under normal conditions. When salt stress is sensed, the interaction between FISL motif and activation loop is relieved, and the kinase activity of SOS2 is released. Analysis of the structure of the SOS2 protein revealed that the FISL domain is found in the region where SOS3 binds to SOS2, and this region is also very conserved (Guo et al., 2001; Chaves-Sanjuan et al., 2014).

#### Regulation of Salt Overly Sensitive Pathway

During the process of individual signal transduction, phosphatase and protein kinase are typically required to repeatedly phosphorylate the target protein in order to transmit information (Liu et al., 2000), so the phosphorylated protein plays a core role in stress signal transduction in plants. Similar to the changes in protein conformation and charge caused by binding of Ca^2+^, phosphorylation provides a negative charge to the protein, while simultaneously changing the protein conformation and interactions with downstream target proteins (Hunter, 1995; Soderling, 1999; Clapham, 2007). A Ca^2+^ signal is converted into phosphorylation according to a step-by-step regulation response (Harmon et al., 2000; Batistic and Kudla, 2004). Salt stress causes changes in intracellular Ca^2+^ concentration. CBLs act as Ca^2+^ receptors, CIPKs act as Ca^2+^ effectors, and CBLs target proteins. Protein kinases form a precise regulatory network under the control of Ca^2+^. The SOS pathway is one of the important targets of CBL/CIPK regulation (Shi et al., 2000; Qiu et al., 2002; Ma et al., 2020; Tang et al., 2020). Other target proteins regulated by CBLs/CIPKs on the plasma membrane include the nitrate ion transporter NRT1.1 (CHL1; Ho et al., 2009), potassium transporter 1 (AKT1; Xu et al., 2006; Lee et al., 2007; Cheong et al., 2010), and potassium transporter 2 (AKT2; Held et al., 2011). The SOS3-SOS2 complex can interact with the low-affinity Na^+^ transporter HKT1 and affect transport of Na^+^ into the cell. Meanwhile, SOS2 can also interact with vacuolar Na^+^/H^+^ transporters (Na^+^/H^+^ exchangers, NHXs) to affect the balance of Na^+^ in plant cells. However, SOS2 does not necessarily have to be activated by SOS3 to form a CBL-CIPK complex and regulate the salt tolerance of plant cells. In other transport systems, such as tonoplast vesicles, SOS2 itself has kinase activity. In the SOS3 non-mutated *sos2 Arabidopsis* mutant, Na^+^/H^+^ transport activity was significantly lower than that of the wild type, and the Na^+^/H^+^ transport activity of the *sos2 Arabidopsis* restored line was reactivated under salt stress (Qiu et al., 2004), demonstrating that SOS2 itself is capable of activating the Na^+^/H^+^ transporter NHX and compartmentalizing Na^+^ into the vacuole. SOS2 serves as an intermediate hub in the SOS signaling pathway. The results of biochemical experiments showed that kinase activity was activated by NaCl induction, but treatment with KCl or mannitol had no significant effect, indirectly suggesting certain specificity in the regulation of SOS1 activity under salt stress (Lin et al., 2009). At the same time, NHX is driven by H^+^-ATPase, and its activity is also directly regulated by SOS2. In addition, the H^+^/Ca^2+^ antiporter CAX also serves as a target protein for SOS2 and does not depend on SOS3, supporting maintained balance of intracellular Ca^2+^.

#### Negative Regulatory Mechanism of SOS Signaling Pathway

Under normal conditions, the 14-3-3 and GIGANTEA (GI) proteins interact with SOS2 to inhibit their kinase activity and inactive the SOS pathway (Kim et al., 2013; Zhou et al., 2014), and their overexpressing plants therefore exhibit a salt-sensitive phenotype (Zhou et al., 2014). Activity of the plasma membrane H^+^-ATPase is negatively affected by SOS3-like calcium-binding protein 1 (SCaBP1)/calcineurin B-like protein 2 (CBL2) and SOS2-like protein kinase 5 (PKS5)/SOS2-like protein kinase 24 (PKS24) regulation. Recent studies have reported that CBL10/SCaBP8 has a similar inhibitory effect on activity of the plasma membrane H^+^-ATPase (Xie et al., 2021). ABA-INSENSITIVE2 (ABI2), a member of the protein phosphatase 2C family, interacts with SOS2 and has the potential to inhibit SOS2 protein kinase activity (Ohta et al., 2003). When the salt stress signal is sensed, both 14-3-3 and GI are degraded by the 26S proteasome pathway, and SOS2 kinase activity is rapidly restored (Kim et al., 2013; Tan et al., 2016). At the same time, the heat shock protein J3 inhibits the protein kinase activity of SCaBP1/PKS5 by binding to PKS5, releasing the plasma membrane H^+^-ATPase (Fuglsang et al., 2007; Yang et al., 2010), which provides a proton potential energy gradient for SOS1’s antiport function. In addition, SCaBP8 can directly interact with the C-terminus of the low-affinity K^+^ channel AKT1 to inhibit K^+^ transport into the cell (Ren et al., 2013). Under salt stress, SOS2 mediates the separation of SCaBP8 from AKT1 through phosphorylation, relieves the inhibitory effect of K^+^ uptake by plants (Lin et al., 2009; Du et al., 2011), and stabilizes the SOS2-SCaBP8 protein complex in the plasma membrane. As a result, SOS2 activates protein activity of AKT1 and SOS1 through phosphorylation regulation with SCaBP8, thereby regulating the dynamic cytoplasmic balance of Na^+^/K^+^ and improving plant salt tolerance.

## Crosstalk in Signaling Pathways

The excretion of Na^+^ in aerial regions is the preferred strategy by which many glycophytes obtain resistance to high-salt environments (Hauser and Horie, 2010). When the SOS signal pathway is activated, the Na^+^/H^+^ antiport activity of SOS1 is enhanced, and accumulated Na^+^ is transported out of the cell. At the same time, the Na^+^/H^+^ antiporter NHX1, found on the vacuole or vesicle membrane, compartmentalizes Na^+^ to the vacuole or vesicles to reduce accumulation of cytosolic Na^+^. Plants accelerate this endocytosis by inducing the accumulation of cytoplasmic vesicles and reducing fusion of the vacuole membrane, which can be caused by increasing cytosolic salt and which represents an excessively sensitive mechanism by which the prevalence of the Na^+^/H^+^ antiporter SOS1 adapts to salt stress (Hamaji et al., 2009; Zhao et al., 2021b). In salt-acclimated tobacco (*Nicotiana tabacum*), vacuolar Na^+^ compartmentalization may be mediated by vesicular transport (Garma et al., 2015). The vesicle transport regulator AtRab7 (AtRabG3e) participates in the regulation of vesicle transport in *A. thaliana* and accelerates the endocytosis of protoplasts, roots, and leaves. Therefore, transgenic plants accumulate vacuolar Na^+^ and exhibit enhanced salt tolerance (Mazel et al., 2004). *Arabidopsis* vesicle-associated membrane protein 711C (VAMP711C) is involved in the docking of vacuolar vesicles under salt stress (Silverstone et al., 2007). Inhibiting the expression of VAMP711C improves salt tolerance (Leshem et al., 2006). PtdIns and its phosphorylated derivatives are involved in intracellular membrane transport and endocytosis (Dong et al., 2011), and the modified protein At5PTase9 is involved in regulating vesicle transport in response to salt stress (Golani et al., 2013).

Salt stress causes a rapid increase in the concentration of free Ca^2+^ in the plant cytoplasm, activating multiple signal pathways to regulate this dynamic balance of ions (Ma et al., 2020). HKT1 is considered to be one of the key factors involved in plant salinity tolerance in response to salt stress (Platten et al., 2006). Tissue-specific expression of the *HKT1* gene, including specific expression of *HKT1* in the pericylindrical sheath or microtubule bundle, can enhance plant salt tolerance on the whole plant level (Moller et al., 2009). Mutation of *HKT1* in the background of salt-sensitive mutants *sos2* and *sos3* can partially restore the salt-sensitive phenotype, indicating that HKT1 may work with the SOS pathway to regulate intracellular Na^+^/K^+^ homeostasis (Rus et al., 2001, 2004). Studies have shown that HKT1 likely unloads Na^+^ from the root to the xylem cytosol of above-ground tissues (Rus et al., 2004; Davoaport et al., 2007), reducing accumulation of Na^+^ in above-ground plant tissues (Ren et al., 2005; Sunarpi et al., 2005; Horie et al., 2009; Moller et al., 2009), maintaining the balance of K^+^/Na^+^, and alleviating the blockage of K^+^ absorption caused by excessive intracellular Na^+^ concentration, ultimately reducing cell damage and growth inhibition (Niu et al., 1995; Tester and Davenport, 2003). In addition, studies have found that mitogen-activated protein kinase (MAPK6/MPK6) can release its activity through phosphorylation of SOS1. This modification process is dependent on the salt stress response mediated by phosphatidic acid (PA; Yu et al., 2010).

In addition, SOS1 responds to salt stress and oxidative stress by interacting with the regulator of oxidative stress response RCD1. It has been found that RCD1 likely exhibits two distinct functions related to salt tolerance, consistent with changes in its subcellular localization pattern (Katiyar-Agarwal et al., 2006). Under normal conditions, the RCD1 protein exists in a reduced mode in which is localized in the nucleus, interacting with transcription factors such as STO and DREB2A, independent of SOS1 (Belles-Boix et al., 2000). Under conditions of salt stress or oxidative stress, the subcellular localization of RCD1 is altered. Some genes related to oxidative stress tolerance were found to be regulated by both RCD1 and SOS1, and RCD1 and SOS1 mutants exhibit additive effects on the salt-tolerant phenotype (Mou et al., 2003; Foyer and Noctor, 2005). Although the regulatory process of RCD1-SOS1 interaction is not fully understood, another function of RCD1, performed near the cytoplasm and at the nuclear periphery, is closely related to the SOS1 interaction. Transport of RCD1 across plasma membrane, mediated by the end of the Na^+^/H^+^ antiporter cytoplasmic domain, likely affects transduction of oxidative stress signaling (Katiyar-Agarwal et al., 2006). The expression of genes involved in scavenging ROS is regulated by RCD1 and SOS1. Altered expression of *ENH1* and *SOD* genes in *rcd1* and *sos1* mutants may, at least in part, explain the mutants’ enhanced tolerance to MV and enhanced sensitivity to H_2_O_2_ (Katiyar-Agarwal et al., 2006). Intracellular oxidative conditions may lead to changes in the formation of intermolecular disulfide bonds or the phosphorylation state of RCD1, enabling export of the RCD1 protein to the cytoplasm or preventing entry of new RCD1 proteins into the nucleus such that RCD1 is allowed to exist not only in the nucleus, but also in the cytoplasm. These studies inform the biochemical mechanisms of SOS1 and RCD1 in oxidative stress tolerance and provide a new reference for the interplay between ion homeostasis and oxidative stress tolerance pathways during plant salt tolerance.

At present, the signal receptor of most upstream of the SOS signal pathway remains uncertain. By screening the salt-stressed phenotypes of receptor-like kinase mutants, the salt-sensitive mutant *gso1* was identified. GSO1 regulates the SOS pathway mediated by SOS2, which is different from the SOS pathway and may be a far upstream regulator in response to salt stress (Chen, 2018). After plants quickly perceive changes in the external environment, they will produce secondary messengers, such as Ca^2+^, inositol phosphate, ROS, and plant hormones in the cytoplasm, and salt stress signal are further decoded and amplified in a stepwise fashion (Xiong et al., 2002; Xiong and Zhu, 2002; Zhu, 2016). This signal cascade involves perception and binding of Ca^2+^ by calmodulin, protein phosphorylation and dephosphorylation, and phospholipid metabolism (Hashimoto and Kudla, 2011; Chele et al., 2021). Furthermore, the final targets of these cascades may be specific transcription factors that further activate the expression of genes related to salt stress. Relevant studies have shown that SOS2 can phosphorylate ethylene-insensitive 3 (EIN3) to enhance expression of its target genes, indicating that SOS2 may be linked to ethylene signaling and response to salt stress (Quan et al., 2017). The SOS pathway is also regulated by ROS signaling (Zhou et al., 2012). Firstly, the stability of *SOS1* mRNA is regulated by ROS stress (Tripathy and Oelmuller, 2012). In addition, SOS2 can interact with CAT2 (catalase 2) and CAT3, suggesting that SOS2 may be a crucial link between the ROS signaling pathway and response to salt stress (Verslues et al., 2007).

## Concluding Remarks

The SOS pathway is ubiquitous in higher plants. Related research reports have compared salt tolerance-related genes in the dicot model plant *Arabidopsis*, the monocot model crop *O. sativa* and wheat (Pardo et al., 2006; Mullan et al., 2007), including genes that control the entry (*HKT1*) and exit (*SOS1*) of Na^+^ and the sequestration of Na^+^ in vacuoles (*NHX1*, *NHX5*, *AVP1*, and *AVP2*), and found that *Arabidopsis* contains genes homologous to *NHX1*, *NHX5*, and *SOS1* in *O. sativa* and wheat. Except for the amino and carboxyl termini, most exons are conserved. However, compared with *Arabidopsis*, the *NHX1* and *SOS1* genes of *O. sativa* and wheat contain extra exons, indicating that the gene sequence of the regulatory region has undergone rearrangement during plant evolution. Studies on the SOS pathway in *O. sativa* and *Arabidopsis* have shown that the regulatory proteins making up the SOS pathway are functionally complementary in these two distant plants. In addition to the interaction of OsCBL4 with OsCIPK24 to regulate the Na^+^/H^+^ exchange activity of OsSOS1, OsSOS1 can be regulated by the AtSOS2 protein kinase alone, while *Arabidopsis* SOS1 cannot be activated by AtSOS2 alone (Martínez-Atienza et al., 2007). Current molecular breeding programs often combine different genes and mutant functional genes that regulate plant salt tolerance, leveraging genetic variation in known salt tolerant mechanisms to improve crop yield (Zhao et al., 2006; Liu et al., 2010; Wu et al., 2015). Based on current broad consensus on the theory of salt tolerance, the CBL10/SOS3-SOS2/CIPK8-SOS1 signaling pathway is the basis for salt tolerance in plants (Quan et al., 2007; Quintero et al., 2011; Yin et al., 2020). This salt-tolerance pathway system was reconstructed in salt-sensitive yeast cells by expressing exogenous SOS pathway regulatory genes. The results showed that transgenic yeasts expressing AtSOS1-Δ998 or SOS2T168D/Δ308 exhibited salt-tolerance advantages (Quintero et al., 2011). Wheat TaSOS1-Δ974 and *Arabidopsis* AtSOS-Δ998 not only performed similarly in enhancing salt tolerance in transgenic yeast cells, but also endowed transgenic plants with obvious salt tolerance (Feki et al., 2014; Zhou et al., 2016). Genetic studies using model plant overexpression lines and knockout mutants have shown that mutations in *CBL10*, *SOS3*, *SOS2*, or *SOS1* render *Arabidopsis* sensitive to salt stress (Wu et al., 1996; Zhu et al., 1998; Quan et al., 2007). Therefore, effective means of genetic engineering can be emphasized to express hyperactive mutant gene, such as AtSOS1-Δ998, AtSOS2T168D/Δ308, and TaSOS1-Δ974 (Feki et al., 2014; Zhou et al., 2016), or co-express the SOS pathway genes to aggregate crop varieties with salinity tolerance (Ma et al., 2014; Zhou et al., 2015; Nutan et al., 2018; Cheng et al., 2019; Fan et al., 2019).

With the deepening of research on the SOS signaling pathway, the Na^+^/H^+^ antiport activity of SOS1 is no longer considered to be single-line signaling pathway, but rather a network of signaling elements, allowing the participation of multiple effecter proteins and regulation of multiple elements in parallel (Ji et al., 2013; Zhao et al., 2020). Therefore, the final output of SOS signaling is likely to be the cumulative effect of multi-dimensional and multi-pathway regulation. The regulatory flexibility of this signaling network is high, allowing plants to respond to specific conditions at the cell, tissue, and organ levels and during different developmental stages, therefore shows a different degree of complexity. However, flexible signaling must involve physical protein molecules and, because SOS1 function is a prerequisite for salt tolerance, these new components must be linked to SOS1 in some way, In the process of studying regulation of the plant stress network, plants are often unexpectedly found to have acquired a certain salt tolerance through deletion of certain genes, potentially exceeding the expected tolerance of the SOS1 overexpression phenotype in terms of salt capacity improvement. Through the continuous in-depth study of the SOS signaling pathway in specific species, we can more comprehensively understand the role of SOS1 in the regulatory network to avoid signaling pathway crosstalk. In systematic studies, these findings are expected to initiate new exploration and understanding in salt stress research, in addition to providing additional references for improving crop tolerance.

## Author Contributions

XJ and YZ conceived and designed the review. QX and XJ wrote the manuscript. All authors contributed to the article and approved the submitted version.

## Funding

This study is supported by National Key R&D Program of China (2018YFE0207203-2, 2018YFD1000500), National Natural Science Foundation of China (31660253), and the Foundation of Sansha City.

## Conflict of Interest

The authors declare that the research was conducted in the absence of any commercial or financial relationships that could be construed as a potential conflict of interest.

## Publisher’s Note

All claims expressed in this article are solely those of the authors and do not necessarily represent those of their affiliated organizations, or those of the publisher, the editors and the reviewers. Any product that may be evaluated in this article, or claim that may be made by its manufacturer, is not guaranteed or endorsed by the publisher.
